# An Optimized Real-Time PCR to Avoid Species-/Tissue-Associated Inhibition for H5N1 Detection in Ferret and Monkey Tissues

**DOI:** 10.1100/2012/907095

**Published:** 2012-05-03

**Authors:** LingJun Zhan, LinLin Bao, FengDi Li, Qi Lv, LiLi Xu, Chuan Qin

**Affiliations:** ^1^Key Laboratory of Human Diseases Comparative Medicine, Ministry of Health, Institute of Laboratory Animal Science, Chinese Academy of Medical Sciences (CAMS) & Comparative Medicine Centre, Peking Union Medical Collage (PUMC), Pan Jia Yuan Nan Li No. 5, Chao Yang District, Beijing 100021, China; ^2^Key Laboratory of Human Diseases Animal Model, State Administration of Traditional Chinese Medicine, Pan Jia Yuan Nan Li No. 5, Chao Yang District, Beijing 100021, China

## Abstract

The real-time PCR diagnostics for avian influenza virus H5N1 in tissue specimens are often suboptimal, since naturally occurring PCR inhibitors present in samples, or unanticipated match of primer to unsequenced species' genome. With the principal aim of optimizing the SYBR Green real-time PCR method for detecting H5N1 in ferret and monkey (Chinese rhesus macaque) tissue specimens, we screened various H5N1 gene-specific primer pairs and tested their ability to sensitively and specifically detect H5N1 transcripts in the infected animal tissues, then we assessed RNA yield and quality by comparing Ct values obtained from the standard Trizol method, and four commonly used RNA isolation kits with small modifications, including Roche High Pure, Ambion RNAqueous, BioMIGA EZgene, and Qiagen RNeasy. The results indicated that a single primer pair exhibited high specificity and sensitivity for H5N1 transcripts in ferret and monkey tissues. Each of the four kits and Trizol reagent produced high-quality RNA and removed all or nearly all PCR inhibitors. No statistically significant differences were found between the Ct values from the isolation methods. So the optimized SYBR Green real-time PCR could avoid species- or tissue-associated PCR inhibition in detecting H5N1 in ferret and monkey tissues, including lung and small intestine.

## 1. Introduction

Ferrets have emerged as an appropriate and feasible model system of influenza, especially for evaluating the efficacy of antiviral drugs and vaccines. In contrast, the monkey is superior for infection and immunity studies since it is more genetically similar to human [1].

Highly pathogenic avian influenza virus (HPAIV) infection normally targets the mucosal tissues but can rapidly spread to multiple organs, eliciting robust cytokine-mediated systemic inflammation, and possible death. Therefore, in addition to oral and cloacal swabs, tissue biopsies are often used to monitor HPAIV infection in infected birds and animals, especially from lower respiratory tract and digestive tract. Viral transcripts can be effectively detected in infected tissues by qPCR, which has been broadly applied to clinical diagnostics, surveillance, and research [1].

However, qPCR detection in tissues sometimes was not ideal. There were several reasons. First, it could be limited by naturally occurring inhibitory substances that were present in some clinical and environmental samples, including feces, blood, soil, tissues, and urine [2, 3]. Such molecules might be coextracted and copurified with the RNA during isolation from the infected tissues and fecal swabs under examination [4]. But commercially available RNA extraction kits might fail to completely remove such amplification inhibitors [5].

In addition, despite the fact that ferrets have been used in biomedical research for decades, little is known about the ferret genome. Thus, it is difficult to design primer to avoid the potential mismatch between primer sequences of pathogen and the whole genome of animals in PCR. This limitation is, unfortunately, not limited to a single animal type and affects many of the nonhuman primates that have not yet to be sequenced.

In our previous study, we determined that the qPCR based on the SYBR Green reagent was ideal for detecting H5N1 from human nasal swabs, ferret or monkey nasal swabs, respiratory tract lavage and turbinate curettage biopsy, and mouse lung tissue (data not shown), but the results from ferret or monkey tissues were suboptimal. We observed nonspecific amplification products and many instances of complete failure of amplification from confirmed infected tissues. In order to identify an effective qPCR system for these two animal species we had to first identify the most optimal primer pair sequences and technique to extract excellent quality RNA.

## 2. Materials and Methods

### 2.1. Reagents

The manufacturer-supplied RNA isolation kits were Qiagen RNeasy mini kit (catalogue #74106; QI), Ambion RNAqueous kit (AM1912; AM), Roche High Pure RNA tissue kit (12033674001; RO), and BioMIGA EZgene tissue RNA miniprep kit (R6311; BI). Invitrogen's Trizol reagent (15596-026; TR) was also used, according to manufacturer's instructions. cDNA was reverse transcribed from isolated total RNA using the SuperScript III first-strand synthesis system (18080-051; Invitrogen). qPCR was carried out using the Power SYBR Green PCR master mix (4367659; Applied Biosystems, Inc.).

### 2.2. Animals

Ferrets (Mustela Pulourius Furo), 4-5 months of age (Marshall Farms,USA), Monkeys (Chinese rhesus macaques), 3-year-old (the Academy of Military Medical Sciences in Beijing). The animals were serologically negative detected by hemagglutination inhibition (HI) assay for currently circulating influenza viruses including A/California/7/2009 (H1N1), seasonal influenza virus H1N1, H3N2, and avian influenza virus H5N1.

### 2.3. Tissue Sample Collection and Homogenization

Organ samples were obtained from experimentally infected ferrets and monkeys, which were nasal swab positive for AIV H5N1 (SZ406H) and experiencing obvious clinical symptoms, such as fever, sneezing, and runny nose. Prior to biopsy, the animals were euthanized by injecting Tribromoethanol. The tissues were ground up by a Pro-200 tissue homogenizer (Pro Scientific) to a homogeneous lysate; solid debris was removed by centrifugation, and the remaining liquid was prepared for virus quantitation by qPCR. All the experiment was carried out in ABSL-3 lab [6], and all procedures were approved by the Institute of Animal Use and Care Committee of the Institute of Laboratory Animal Science, Peking Union Medical College (MC-09-7005).

### 2.4. RNA Isolation from Ferret and Monkey Tissues

The commercial kits and reagents were used applied with the manufacturer's recommendation, including any modifications introduced [6]. The starting material for all procedures was 50 *μ*L homogeneous tissue sample (~10 mg instead of 30 mg), and the samples were transferred to its starting buffer (e.g., RLT buffer for QI kit) with volume of 500 *μ*L, respectively. At the last step, the isolated RNA was resolved in RNase-free water or Elution buffer with final volume of 50 uL. The manufacturer's protocols were almost completely followed except some small modifications. As for QI kit, after step 7, 50 *μ*L DNase was added per isolation column and incubated for 1 min at room temperature.

Total RNA was transcribed with SuperScript III First-Strand synthesis system, and the virus-containing supernatant of infected Madin-Darby Canine Kidney (MDCK) epithelial cells was purified and also transcribed as a positive control.

### 2.5. Primer Design

Primer pairs were designed for the eight gene fragments of H5N1, three pairs for each gene (Table 1). In addition, primer pairs that recommended by WHO were synthesized and tested (Table 2).

### 2.6. Primer Screening for SYBR Green Real-Time PCR of Ferret and Monkey Tissues

The primer pairs listed in Tables 1 and 2 were used to amplify the cDNA of H5N1 virus (SZ406H, Genebank ID: 133711835), and several of the most superior primer pairs were selected for further analysis. The selected primer pairs were then used to amplify cDNAs of infected lung and small intestine, as prepared by the five commercial kits, by SYBR Green qPCR; the primer pair exhibiting the highest specificity and sensitivity was selected from the set. The primer pair was also chosen based upon its suitability in both ferret and monkey tissues. The sensitivity of this primer pair in detection was then evaluated with serial standard samples having known copy numbers of virus (from 10 to 10^8^). DNA sequence of some PCR products was confirmed by sequencing (Taihe Biotechnology Co., Ltd.).

### 2.7. Comparative Analysis of the Commercial RNA Isolation Kits and TR in Ferret and Monkey Tissues

The quality of different RNA isolation kits and the TR method was evaluated for their ability to remove PCR inhibitors from the complex tissue samples. The Ct value obtained from amplifying the same amount of tissue template was compared among all five methods [6–8].

## 3. Results

### 3.1. Identification of an Optimal Primer Pair for SYBR Green Real-Time PCR-Based Detection of H5N1 in Ferret and Monkey Tissues

**Figure 1:**
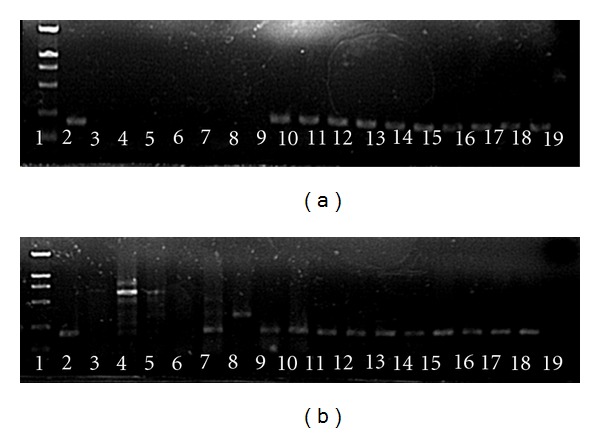
Agarose gel electrophoresis of SYBR Green real-time PCR products of H5N1 transcripts in lung and small intestine tissues. Lane 1: DL2000 Marker; Lane 2: positive control; Lanes 3–5: small intestine samples amplified with primer pairs N1-1/N1-2, M30F/M264R, H5-1/H5-3; Lanes 6–8: lung samples amplified with primer pairs N1-1/N1-2, M30F/M264R, H5-1/H5-3; Lanes 9–13: small intestine samples prepared by TR, QI, BI, AM, and RO, respectively, and amplified by primer pair SZNP-F2/R2; Lanes 14–18: lung samples prepared by TR, QI, BI, AM, and RO, respectively, and amplified with primer pair SZNP-F2/R2; Lane 19: negative control. (a) was for ferret tissues and (b) was for monkey tissues.

A group of candidate primer pairs for eight H5N1 genes was screened for specificity and sensitivity of detection of positive virus template by PCR and agarose gel electrophoresis. The screened primers included SZHA-F1/R1, SZNA-F2/R2, SZM-F2/R2, SZNP-F2/R2, SZNS-F2/R2, SZPA-F1/R1, SZPB1-F3/R3, and SZPB2-F2/R2 (see Table 1). The primer pair SZNP-F2/R2 among them exhibited the highest specificity and sensitivity and performed better than the WHO recommended primer pairs H5-1/H5-3, M30F/M264R, and N1-1/N1-2 (Figures 1(a) and 1(b)).

### 3.2. The Specificity and Sensitivity of Primers SZNP-F2/R2 in Ferret and Monkey Tissues

**Figure 2:**
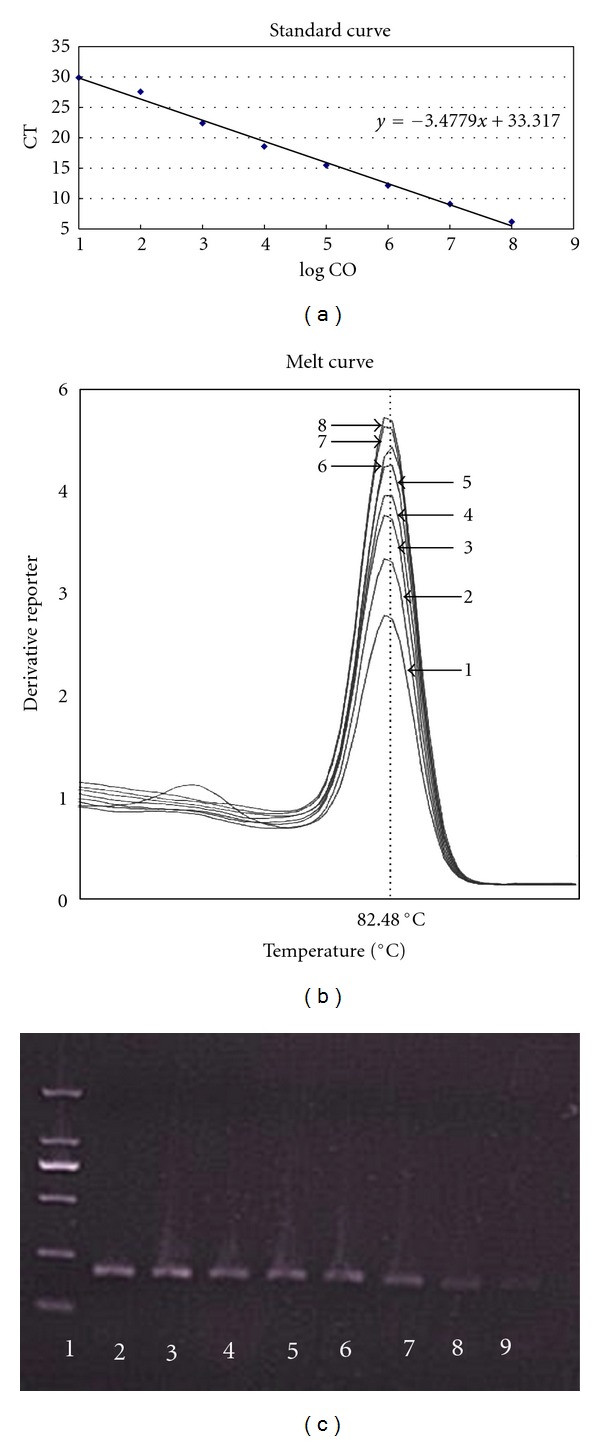
The sensitivity and specificity achieved with primer pair SZNP-F2/R2 in SYBR Green real-time PCR. (a) Standard curve. LogCO: log10 (copies), slope: −3.4779, *R* ^2^: 0.998, eff%: 110%. (b) Melting curve. (c) Agarose electrophoresis of PCR products of H5N1. Lane 1: DL2000 Marker; Lanes 2–9: 10^8^–10^1^ copies of H5N1 virus cDNA.

cDNAs of uninfected ferret and monkey tissues mixed with a range of virus (copy number from 10 to 10^8^) were used to evaluate the SZNP-F2/R2 primer pair in SYBR Green qPCR. According to the standard curve, the linear equation for the qRT-PCR was *Y* = −3.4779*X* + 33.317. The sensitivity of this method, defined as the lowest concentration of cDNA detected in qPCR, was 1 copy/reaction (Figure 2(a)). Moreover, the SZNP-F2/R2 specificity was high, as evidenced by the characteristic monowave profile of the melting curve and the presence of a single band in agarose gel electrophoresis (Figures 2(b) and 2(c)). The DNA sequence of qPCR product was also exactly the same as the fragment 1123–1423 bp of SZNP (Genebank ID: 133711835), as confirmed by DNA sequencing.

### 3.3. The Ct Values for the Five RNA Isolation Methods Used in Conjunction with SYBR Green Real-Time PCR of Ferret or Monkey Tissues

cDNAs prepared from RNA isolated by the five different methods were amplified by SYBR-Green qPCR. The respective Ct values from each, using equal amounts of sample material, were listed in Table 3. The melting curves and the agarose gel electrophoresis results were shown in Figures 1(a) and 1(b). The results indicated that the four commercially available kits and the Trizol reagent were sufficiently effective to produce high-quality RNA suitable for H5N1 transcripts detection in ferret and monkey tissues. We concluded that the five methods were comparable in removing potentially contaminating PCR inhibitors from tissue samples; our findings in small intestine were particularly insightful since this tissue harbors a complex enzyme profile [6].

## 4. Discussion

The WHO recommended primer pairs were designed to target Clade 1, 2, and 3 H5N1 viruses from respiratory biopsies, lavage, and swab specimens. However, we found that they were not suitable for detecting H5N1 in ferret and monkey tissues (Figure 1), which may be the result of unanticipated match to sequence of species- and/or tissue-specific cDNAs. The yet undefined whole genomes of ferret and Chinese rhesus macaque (also other macaques) made screening for appropriate and effective H5N1 primers challenging; thus, we designed a group of candidate primers and sought to identify a single pair with the highest sensitivity and specificity for H5N1 virus in these animals. The primer pair SZNP-F2/R2 fit these qualifications and avoided mismatches between primer and tissues cDNA sequences from either ferret or monkey. In addition, SZNP-F2/R2 was able to properly detect H5N1 in nasal swabs and in supernatants from virus-infected cultured cells by using SYBR-Green qPCR and conventional PCR. Therefore, our newly designed SZNP-F2/R2 primers represent the first reported primer pair that was applicable for H5N1 detection in both human swab samples (data not shown) and ferret or monkey samples, including swab, lavage, and tissue specimens.

Numerous commercial kits for total RNA extraction are available, the QI RNeasy mini kit has been reported as sufficient for obtaining RNA from a wide variety of tissue samples and from viruses suspended in culture supernatants [1]. While it has also been reported that the silicon nucleic acid binding column method is not effective when using tissues, cloacal swabs or samples that may contain fecal material; instead, the organic extraction method with Trizol reagent is recommended for use with amnioallantoic fluid samples, oral swabs, tracheal swabs, cloacal swabs, cell culture material, and tissues with complex enzyme profiles [8]. Meanwhile, since the current commercially available column-based RNA isolation kits were developed and optimized using mouse and rat tissue, we needed to determine whether they worked as effectively with ferret and monkey tissues. It is essential to choose a proper RNA isolation method for our own experiment according to different tissues and real-time PCR techniques being used.

In this study, we compared five commonly used RNA isolation methods with some modifications, including QI, AM, RO, BI, and TR. After comparing the Ct value of samples prepared by each, we found that all were capable of sufficiently isolating high quality RNA, without significant statistical differences in yield or quality. Moreover, each method appeared to effectively remove the endogenous PCR inhibitors from the tissues examined; this is an especially insightful finding as the small intestine tissue is presumed to contain a large profile of various enzymes and potential PCR inhibitors [5, 7]. Therefore, the five RNA isolation methods are all effective for viral RNA isolation from tissues of ferret and monkey, and for subsequent detection of H5N1 by SYBR-Green qPCR.

 The optimized SYBR-Green qPCR system, though it has many advantages, still has some limitations that must be taken into consideration. The melting curve might sometimes mask nonspecific products; hence, it was recommended to verify the real time PCR products by agarose gel electrophoresis to confirm single- or multiple-band products in preliminary study. Moreover, due to the conservation of NP in influenza virus nucleotide sequence, the primer SZNP-F2/R2 might match with two AIV H9N2 strains by BLAST analysis, which needed further more experimental confirmation. But it was confirmed that the SZNP-F2/R2 was perfect in detecting H5N1 in experimental-infected ferret and monkey tissues.

In conclusion, the findings from this study provide insights into the necessary steps and reagents that will help to resolve the current problems experienced when using SYBR-Green qPCR to detection H5N1 in particular tissues. It is important to consider the possibility of nonspecific amplification from different species and tissues, which may be a result of mismatch or unanticipated extra matching of primer and particular AIV strain or animal genome, respectively. It is also important to determine if failed amplifications are a result of inhibition by contaminating PCR inhibitors in samples. This qPCR-based viral detection is applicable for other viruses and nonviral pathogens detection in a wide array of tissues.

## Figures and Tables

**Table 1:** Primer pairs for H5N1 (SZ 406H).

Target gene	Primer name	Sequence (5′-3′)
HA	SZHA-F1/R1	CCATTCCACAACATACACCCTC/TTCCCTGCCATCCTCCCT
	SZHA-F2/R2	ACAAGGTCCGACTACAGC/TTCCGTTTCTTACACTTTCC
	SZHA-F3/R3	AGAACAATACATACCCAACA/CACTTTGCCCGTTTACTT

NA	SZNA-F1/R1	CAAAGACAGAAGCCCTC/CTCAGTATGTTGTTCCTCCA
	SZNA-F2/R2	ACAGGGAATCAACACCAA/TACAGCCCATCCTCTAAT
	SZNA-F3/R3	AAGACAGAAGCCCTCACAG/TTTCAATACAGCCACAGCC

NP	SZNP-F1/R1	TCAGCGTTCAGCCCACTT/TCGGGTTCGTTGCCTTTT
	SZNP-F2/R2	CAGCCCACTTTCTCGGTAC/TCGGGTTCGTTGCCTTTT
	SZNP-F3/R3	GCCAGGTCTTTAGTCTCAT/CTTATAGCCCAATATCTACTTC

NS	SZNS-F1/R1	AATGCCGACTTCACGCTAC/TCCCACGATTGCTCCTTC
	SZNS-F2/R2	ATGCCCAAGCAGAAAGTG/TCCGATGAGGACGCCAAT
	SZNS-F3/R3	AAGAAGGAGCAATCGTGG/CGTTTCTGATTTGGAGGG

M	SZM-F1/R1	ATTTGTATTCACGCTCACC/TAGTCACCGTTCCCATCC
	SZM-F2/R2	TTTTGTCCAGAATGCCCTAA/CACCGTTCCCATCCTGTT
	SZM-F3/R3	TACAACAGGATGGGAACG/AGTGGGTTGGTGATGGTT

PA	SZPA-F1/R1	GGAGTGACACGGAGGGAA/TCTCGGATTGACGAAAGG
	SZPA-F2/R2	TGGGATTCCTTTCGTCAA/CTGGAGAAGTTCGGTGGG
	SZPA-F3/R3	TCTATGGGATTCCTTTCG/TCTGGCGTTCACTTCTTT

PB1	SZPB1-F1/R1	CTTGAAGAATCCCACCCA/AAATCTATCAGCCGTCCC
	SZPB1-F2/R2	ACATACCGATGCCACAGA/TCAATTCCCATTTCAAGC
	SZPB1-F3/R3	GCGAGGAGTATCTGTGAG/ATCATTGCCAGAAACATC

PB2	SZPB2-F1/R1	CTGGGTCGGACAGGGTGAT/AACTCGGCGGCGTATTTT
	SZPB2-F2/R2	AAACTGGGAGACCGTGAA/GCTCTGCTTAGGTGGTGC
	SZPB2-F3/R3	AAACTGGGAGACCGTGAA/CTGCTCTGCTTAGGTGGT

**Table 2:** Primer pairs recommended by WHO for H5N1.

Target gene	Primer name	Sequence (5′-3′)
HA	H5-1/H5-3	GCCATTCCACAACATACACCC/CTCCCCTGCTCATTGCTATG
M	M30F/M264R	TTCTAACCGAGGTCGAAACG/ACAAAGCGTCTACGCTGCAG
N1	N1-1/N1-2	TTGCTTGGTCGGCAAGTGC/CCAGTCCACCCATTTGGATCC

**Table 3:** Ct values of ferret or monkey cDNAs prepared by five different methods and subjected to SYBR-Green qPCR.

	RO	AM	BI	QI	TR
Ferret lung	20.51	18.54	18.85	17.99	17.31
Ferret small intestine	18.29	18.36	18.31	18.29	18.25
Monkey lung	18.31	18.39	18.58	18.34	18.43
Monkey small intestine	20.11	20.26	19.32	17.05	20.18

Note: The Ct values shown are the average of at least 3 replicates with standard deviations of 6.0–10%. The *P* values from *t*-test calculations were >0.05 between the different methods.

## References

[1] Matsuoka Y, Lamirande EW, Subbarao K (2009). The ferret model for influenza. *Current Protocols in Microbiology*.

[2] Rådström P, Knutsson R, Wolffs P, Lövenklev M, Löfström C (2004). Pre-PCR processing: strategies to generate PCR-compatible samples. *Applied Biochemistry and Biotechnology—Part B*.

[3] Suarez DL (2008). *Avian Influenza Virus RNA Extraction from Tissue and Swab Material*.

[4] Liu S, Hou G, Zhuang Q (2009). A SYBR Green I real-time RT-PCR assay for detection and differentiation of influenza A(H1N1) virus in swine populations. *Journal of Virological Methods*.

[5] Tomaso H, Kattar M, Eickhoff M (2010). Comparison of commercial DNA preparation kits for the detection of Brucellae in tissue using quantitative real-time PCR. *BMC Infectious Diseases*.

[6] Das A, Spackman E, Pantin-Jackwood MJ, Suarez DL (2009). Removal of real-time reverse transcription polymerase chain reaction (RT-PCR) inhibitors associated with cloacal swab samples and tissues for improved diagnosis of Avian influenza virus by RT-PCR. *Journal of Veterinary Diagnostic Investigation*.

[7] Das A, Spackman E, Thomas C, Swayne DE, Suarez DL (2008). Detection of H5N1 high-pathogenicity avian influenza virus in meat and tracheal samples from experimentally infected chickens. *Avian Diseases*.

[8] Das A, Spackman E, Senne D, Pedersen J, Suarez DL (2006). Development of an internal positive control for rapid diagnosis of avian influenza virus infections by real-time reverse transcription-PCR with lyophilized reagents. *Journal of Clinical Microbiology*.

